# MicroRNA-495 inhibits the high glucose-induced inflammation, differentiation and extracellular matrix accumulation of cardiac fibroblasts through downregulation of NOD1

**DOI:** 10.1186/s11658-018-0089-x

**Published:** 2018-05-09

**Authors:** Xiaowei Wang, Haiying Jin, Shifeng Jiang, Yanlan Xu

**Affiliations:** Department of Geriatrics, Qingpu Branch of Zhongshan Hospital Affiliated to Fudan University, Qingpu District, No.1158, Park East Road, Shanghai, 201707 People’s Republic of China

**Keywords:** MicroRNA-495, Human cardiac fibroblasts, High glucose, Cardiac fibrosis, NOD1

## Abstract

**Background:**

MicroRNAs (miRNAs) have physiological and pathophysiological functions that are involved in the regulation of cardiac fibrosis. This study aimed to investigate the effects of miR-495 on high glucose-induced cardiac fibrosis in human cardiac fibroblasts (CFs) and to establish the mechanism underlying these effects.

**Methods:**

Human CFs were transfected with an miR-495 inhibitor or mimic and incubated with high glucose. The levels of NOD1 and miR-495 were then determined via quantitative RT-PCR. Pro-inflammatory cytokine levels, cell differentiation and extracellular matrix accumulation were respectively detected using ELISA, quantitative RT-PCR and western blot assays. The luciferase reporter assay, quantitative RT-PCR and western blot were used to explore whether NOD1 was a target of miR-495. The effects of miR-495 on the NF-κB and TGF-β1/Smad signaling pathways were also detected via western blot.

**Results:**

Our results show that high glucose can significantly increase the expression of NOD1 in a time-dependent manner. Upregulation of miR-495 significantly alleviated the high glucose-induced increases in cell differentiation and collagen accumulation of CFs. Moreover, the bioinformatics analysis predicted that NOD1 was a potential target gene for miR-495. The luciferase reporter assay showed that miR-495 can directly target NOD1. The introduction of miR-495 could significantly inhibit the high glucose-activated NF-κB and TGF-β1/Smad signaling pathways.

**Conclusion:**

Upregulation of miR-495 ameliorates the high glucose-induced inflammatory, cell differentiation and extracellular matrix accumulation of human CFs by modulating both the NF-κB and TGF-β1/Smad signaling pathways through downregulation of NOD1 expression. These results provide further evidence for the protective effect of miR-495 overexpression in cases of high glucose-induced cardiac fibrosis.

## Background

Diabetes mellitus is a global health concern, in part due to the associated increased risk of cardiovascular disease [1]. Cardiac fibrosis is a key pathogenic component of cardiovascular diseases [2]. It is characterized by excessive synthesis and pathological deposition of extracellular matrix (ECM) proteins in cardiac tissue, which contributes to cardiac dysfunction and heart failure [3]. However, no treatment for cardiac fibrosis has been found thus far.

Cardiac fibroblasts (CFs) are reported to play the most important roles in cardiac fibrosis as they are involved in the collagen synthesis and deposition. Increased collagen deposition results in more severe fibrosis [4]. High glucose promotes collagen production and eventually contributes to cardiac dysfunction [5]. A viable strategy for treating cardiac fibrosis might be to inhibit the activation of CFs. However, the precise mechanisms underlying high glucose-induced cardiac fibrosis remain unknown.

Nucleotide-binding oligomerization domain-containing protein 1 (NOD1) participates in multiple pathological processes, including tumor development and septic shock, and plays important roles in the pathogenesis of diabetes in adipose, liver and cardiac tissues [6–10]. However, the mechanisms underlying these actions remain unclear.

NOD1 is expressed in the heart and its selective activation is functional in both the cardiomyocyte and CF populations [11]. A previous study showed that NOD1 is overexpressed in the murine and human myocardium in cases of type 2 diabetes mellitus [7]. Moreover, Val-Blasco et al. found that activation of NOD1 modulated cardiac fibrosis is closely associated with diabetic cardiomyopathy using a genetic murine model of type 2 diabetes mellitus [3]. The high levels of NOD1 in CFs were observed in cardiac human necropsies of type 2 diabetes mellitus patients [3], supporting the animal model results. Which miRNA regulates the expression of NOD1 regulated remains unknown.

MicroRNAs (miRNAs) are a type of endogenous small noncoding RNA that regulate targeted gene expression by binding to complementary sequences in the 3′-untranslated region (3’-UTR) at the post-transcriptional level [12]. Recent studies have shown that miRNAs are involved in the regulation of cardiac fibrosis [13–16]. The precise molecular mechanisms and functional role of miR-495 in high glucose-induced cardiac fibrosis remain unclear. In our study, introduction of miR-495 had a protective effect on CFs that were exposed to high glucose, reducing pro-inflammatory cytokines, cell differentiation and extracellular matrix accumulation. We found that NOD1 is a direct target of miR-495 in CFs. Our results also showed that overexpression of miR-495 significantly inhibits the high glucose-induced NF-κB and TGF-β1/Smad signaling pathways by downregulating NOD1 expression.

This shows that miR-495 plays critical roles in the pathogenesis of diabetic cardiac fibrosis and suggests that it may have applications in the treatment of cardiac fibrosis in patients with diabetes mellitus.

## Methods

### Cell culture, transient transfection and glucose treatments

Human CFs were purchased from ScienCell, and cultured in fibroblast medium-2 containing 5% fetal bovine serum (FBS; GIBCO), 1% penicillin/streptomycin (GIBCO), and 1% fibroblast growth supplement-2 (ScienCell) at 37 °C in 5% CO_2_ on 0.1% gelatin-coated culture flasks. Human CFs from passages 3 to 5 were used for our experiments.

The miR-495 inhibitor, miR-495 mimic, miR-negative control for the inhibitor (miR-NC inhibitor), miR-negative control for the mimic (miR-NC) were synthesized and purified by RiboBio. The miR-495 inhibitor (100 nM), mimic (50 nM), miR-NC inhibitor (100 nM) and miR-NC (50 nM) were transfected into CFs using Lipofectamine 3000 reagent (Invitrogen) according to the manufacturer’s protocols.

Transfected and control human CFs were then incubated with normal (5.5 mM) and high (25 mM) glucose for 6, 12, 24 or 48 h. All subsequent assays were performed on cells from one or more of these incubation times.

### RNA extraction and RT-PCR

Total RNA was isolated using Trizol as per the manufacturer’s instructions. An analysis of mRNA levels was performed on a 7500 Fast Real-Time PCR System (Applied Biosystems) with SYBR Green-based real-time PCR for all genes. For miRNA quantification, reverse transcription to cDNA and real-time PCR were performed using an miRNA Extraction Kit (Tiangen) and Taqman miRNA assays (Life Technologies) according to the manufacturers’ instructions. U6 was used as the internal control.

The gene expressions of NOD1, NOS2, COX2, α-SMA, and collagen I and III were detected using the SYBR Green PCR Kit (Qiagen). GAPDH served as an internal control. The following primers were used:

NOD1 forward, 5’-ACTGAAAAGCAATCGGGAACTT-3′, reverse, 5’-CACACACAATCTCCGCATCTT-3’.

NOS2 forward, 5’-AGGGACAAGCCTACCCCTC-3′, reverse, 5’-CTCATCTCCCGTCAGTTGGT-3’.

COX2 forward, 5’-CGCACTTATACTGGTCAAATCCC-3′, reverse, 5’-GCTCAGCAGTAGTAACGAAGGA-3′.

α-SMA forward, 5’-CTATGAGGGCTATGCCTTGCC-3′, reverse, 5’-GCTCAGCAGTAGTAACGAAGGA-3′;

Collagen I forward, 5’-ACGCATGAGCCGAAGCTAAC-3′, reverse, 5’-AGGGACCCTTAGGCCATTGT-3’.

Collagen III forward, 5’-ATAGACCTCAAGGCCCCAAG-3′, reverse, 5’-CCACCCATTCCTCCGACT-3’.

GAPDH forward, 5’-ACAACTTTGGTATCGTGGAAGG-3′, reverse, 5’-GCCATCACGCCACAGTTTC-3′.

### Western blot analysis

The protein used for western blotting was extracted using radio immunoprecipitation assay (RIPA) lysis buffer (Beyotime Biotechnology) supplemented with protease inhibitors (Roche). The proteins were quantified using the BCA Protein Assay Kit (Pierce). The western blot system was established using a Bio-Rad Bis-Tris Gel system according to the manufacturer’s instructions.

Primary antibodies of NOD1 (ab170547; Abcam), p-IKK (#2697), t-IKK (#11930), p-IκBα (#2859), t-IκBα (#4814), p-NF-κB (#3033), t-NF-κB (#8242), p-Smad3 (#9520), t-Smad3 (#9523) and PAI-1 (#11907; Cell Signaling Technology) were prepared in 5% blocking buffer at a dilution of 1:1000. Primary antibodies were incubated with the membrane at 4 °C overnight, followed by washing and incubation with secondary antibody (1:5000, Abcam) marked by horseradish peroxidase for 1 h at room temperature.

After rinsing, the polyvinylidene difluoride (PVDF) membrane-carried blots and antibodies were transferred into the Bio-Rad ChemiDoc XRS system, and then Immobilon Western Chemiluminescent HRP Substrate (Millipore) was added to cover the membrane surface. The signals were captured and the intensity of the bands was quantified using Image Lab Software (Bio-Rad).

### Measurement of NOS2, COX2, TGF-β1, MMP-2, MMP-9 and TIMP-1 levels

As in a previous study [17], the supernatants of CFs were collected after treatment, and the concentrations of NOS2, COX2, TGF-β1, MMP-2, MMP-9 and TIMP-1 were measured using a sandwich ELISA kit (R&D Systems) according to the manufacturer’s instructions. Briefly, the primary antibody was coated onto ELISA plates and incubated for 2 h at room temperature. Samples and standards were added to the wells and incubated for 1 h. Then the wells were washed and a biotinylated antibody was added for 1 h. The plates were washed again and streptavidin conjugated to horseradish peroxidase was added for 10 min. After washing, tetramethylbenzidine was added for color development and the reaction was terminated with 1 mol/l H_2_SO_4_. Absorbance was measured at 490 nm using an automated ELISA reader (Thermo Fisher Scientific). Values were expressed as ng/ml.

### Dual-luciferase reporter assay

The 3’ untranslated region (3’UTR) target site was generated via PCR. The luciferase reporter constructs with the NOD1 3’UTR carrying a putative miR-495-binding site into the pMiR-report vector were amplified via PCR. Cells were co-transfected with the reporter construct, control vector or miR-495 mimic, or the corresponding controls using Lipofectamine 3000 (Invitrogen). Reporter assays were done using the dual-luciferase assay system (Promega) following the manufacturer’s information.

### Statistical analysis

Data are presented as the means ± standard error of the mean (SEM) from at least three independent experiments. All statistical analyses were performed using GraphPad Prism version 6.0 (GraphPad). Student’s t test was used to estimate statistical significance values. Differences were considered to be statistically significant for values of *p* < 0.05.

## Results

### The expression of NOD1 was significantly higher and the level of miR-495 was significantly lower in human CFs incubated with high glucose

**Fig. 1:**
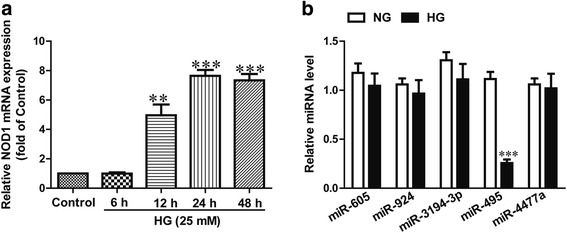
The expressions of NOD1 and miR-495 in human CFs incubated with normal (5.5 mM) or high (25 mM) glucose for 6, 12, 24 or 48 h. **a** The mRNA expression of NOD1 was detected via quantitative RT-PCR. HG indicates CFs treated with 25 mM glucose for the indicated times. **b** The levels of miR-605, miR-924, miR-3194-3p, miR-495 and miR-4477a were determined via quantitative RT-PCR. The data shown are means ± SEM (*n* = 6). NG indicates normal glucose, HG indicates high glucose, time in all cases was 24 h. **p < 0.01, ***p < 0.001 vs. control or normal glucose

To study whether NOD1 was associated with diabetic cardiac fibrosis in vitro, we determined the expression of NOD1 in human CFs incubated with high and normal glucose for 6, 12, 24 or 48 h. Our results showed that the expression of NOD1 was significantly higher in human CFs incubated with high glucose than with normal glucose, and that the increase was time-dependent (Fig. 1a).

Subsequently, the online database (TargetScan 6.2) predicted several miRNAs that could directly target NOD1, including miR-605, miR-924, miR-3194-3p, miR-495 and miR-4477a. Our data indicate that the level of miR-495 was significantly lower in the human CFs incubated with high glucose, but other miRNAs levels did not change (Fig. 1b). These findings suggest that the low level of miR-495 is closely related to the high expression of NOD1 in human CFs incubated with high glucose.

### The effects of miR-495 on pro-inflammatory cytokines in human CFs incubated with high glucose

**Fig. 2:**
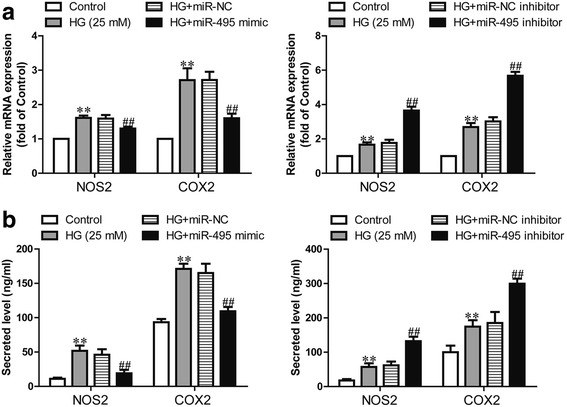
Effects of miR-495 on pro-inflammatory cytokines in human CFs incubated in high glucose. CFs were transfected with miR-495 inhibitor or mimic for 48 h, and then treated with 5.5 or 25 mM glucose for 24 h. HG + miR-NC indicates CFs transfected with the miR-495 inhibitor before treatment with high glucose; HG + miR-495 mimic indicates CFs transfected with the miR-495 mimic before treatment with high glucose. **a** The mRNA expressions of NOS2 and COX2 were determined via quantitative RT-PCR. **b** The secreted NOS2 and COX2 proteins in the supernatants of CFs were detected using ELISA assays. The data shown are means ± SEM (*n* = 4). **p < 0.01 vs. control; ^##^*p* < 0.01 vs. vehicle + high glucose. The vehicle is transfection reagent alone

To study the functional role of miR-495 in the regulation of pro-inflammatory cytokines affected by high glucose, we determined the mRNA and protein expressions of NOS2 and COX2 using quantitative RT-PCR and ELISA assays in high glucose-stimulated human CFs. We found that high glucose could induce the expressions of NOS2 and COX2 in CFs. As expected, high glucose-induced upregulation of NOS2 and COX2 was markedly attenuated in human CFs overexpressing miR-495 (Fig. 2a, b). Furthermore, high glucose-induced upregulation of NOS2 and COX2 were significantly further increased in CFs transfected with miR-495 inhibitor (Fig. 2a, b).

### Effect of miR-495 on high glucose-induced differentiation of CFs into myofibroblasts

**Fig. 3:**
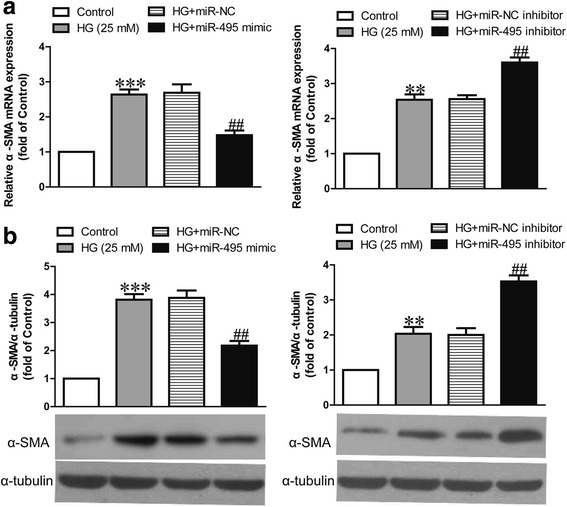
Effects of miR-495 on high glucose-induced differentiation in human CFs. CFs were transfected with miR-495 inhibitor or mimic for 48 h, and then treated with 5.5 or 25 mM glucose for 24 h. HG + miR-NC indicates CFs transfected with the miR-495 inhibitor before treatment with high glucose; HG + miR-495 mimic indicates CFs transfected with the miR-495 mimic before treatment with high glucose. **a** and **b** The mRNA and protein expressions of α-SMA were respectively determined via quantitative RT-PCR and western blot. The data shown are means ± SEM (*n* = 6). **p < 0.01, ***p < 0.001 vs. control; ^##^*p* < 0.01 vs. vehicle + high glucose. The vehicle is transfection reagent alone

To explore the effect of miR-495 on the differentiation of human CFs into myofibroblasts in vitro, we treated human CFs with high glucose for 24 h after transfection with the miR-495 mimic or inhibitor. The mRNA and protein expressions of α-SMA, a myofibroblast marker, in human CFs significantly increased after high glucose stimulation when compared with the control group (Fig. 3). Upregulation of miR-495 evidently inhibited the increase in high glucose-induced α-SMA expression at the mRNA and protein levels (Fig. 3a). Furthermore, miR-495 downregulation promoted high glucose-stimulated expressions of α-SMA (Fig. 3b). These findings indicate that miR-495 overexpression prevents high glucose-induced differentiation of CFs.

### Effect of miR-495 on high glucose-induced imbalance of MMP-TIMP and collagen synthesis in human CFs

**Fig. 4:**
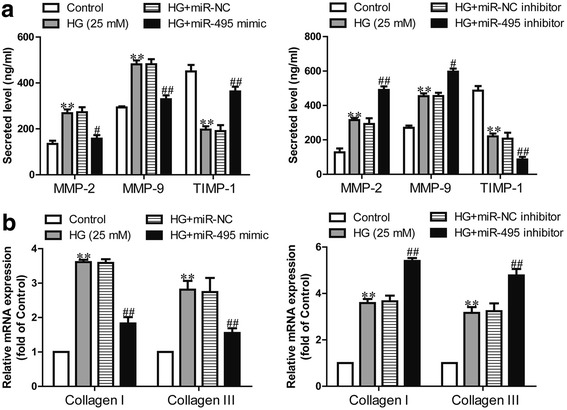
Effects of miR-495 on high glucose-induced imbalance of MMP-TIMP and collagen synthesis in human CFs. CFs were transfected with miR-495 inhibitor or mimic for 48 h, and then treated with 5.5 or 25 mM glucose for 24 h. HG + miR-NC indicates CFs transfected with the miR-495 inhibitor before treatment with high glucose; HG + miR-495 mimic indicates CFs transfected with the miR-495 mimic before treatment with high glucose. **a** The secreted MMP-2, MMP-9 and TIMP-1 proteins in the supernatants of CFs were detected using ELISA assays. **b** The mRNA expressions of collagen I and III were determined via quantitative RT-PCR. The data shown are means ± SEM (*n* = 6). **p < 0.01, vs. control; ^#^*p* < 0.05, ^##^*p* < 0.01 vs. vehicle + high glucose. The vehicle is transfection reagent alone

To gain further insights into the potential roles of miR-495 in cardiac fibrosis, we determined the MMP expressions and collagen synthesis in the supernatants of CFs. The results showed that high glucose meant higher MMP-2 and MMP-9 expressions in the supernatants of CFs than in those of normal glucose group. This level was dramatically decreased by overexpression of miR-495 (Fig. 4a). Moreover, TIMP-1 expression in the supernatants of CFs was downregulated after high glucose treatment, while the miR-495 mimic blocked the high glucose-induced reduction in TIMP-1 expression (Fig. 4a). In line with the altered MMP expressions, the mRNA expressions of collagen I and III were significantly higher in the high glucose group, whereas introduction of miR-495 inhibited the effect of high glucose (Fig. 4b). However, the miR-495 inhibitor could further increase the high glucose-induced upregulation of MMP-2, MMP-9, collagen I and collagen III expressions and downregulation of TIMP-1 expression (Fig. 4a, b).

### miR-495 can directly target NOD1 in HCFs

**Fig. 5:**
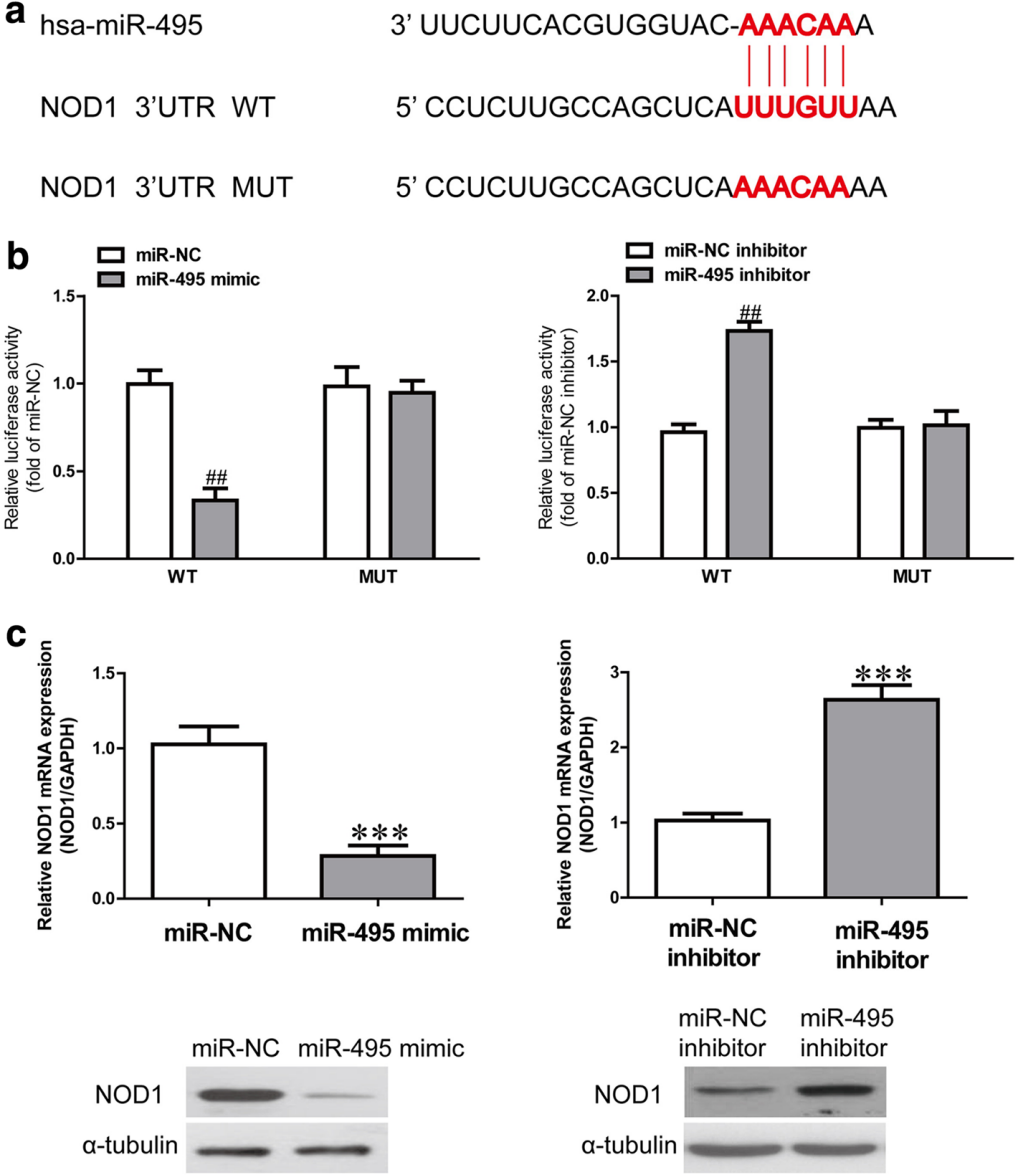
NOD1 was a direct target of miR-495. Human CFs were transfected with miR-495 inhibitor or mimic for 48 h. **a** Schematic representation of NOD1 3’UTRs showing putative miRNA target site. **b** The analysis of the relative luciferase activities of NOD1-WT, NOD1-MUT in CFs. **c** The mRNA and protein expressions of NOD1 were respectively determined via quantitative RT-PCR and western blot. NOD1 expression was normalized to GAPDH. All data are presented as means ± SEM (*n* = 6). ^##^*p* < 0.01, ***p < 0.001 vs. miR-NC or miR-NC inhibitor

The online database TargetScan 6.2 was used to identify an miR-495-binding site in the 3’UTR of NOD1 (Fig. 5a). To validate whether NOD1 is a direct target of miR-495, luciferase plasmids containing the potential NOD1 miR-495-binding sites (WT) or a mutated NOD1 3’UTR were constructed (Fig. 5a). Overexpression of miR-495 inhibited WT NOD1 reporter activity but not the activity of the mutated reporter construct in human CFs, showing that miR-495 could specifically target the NOD1 3’UTR by binding to the seed sequence (Fig. 5b). Next, we confirmed the results at the mRNA and protein levels. Introduction of miR-495 significantly decreased the expression of NOD1, whereas knockdown of miR-495 increased the NOD1 expression in CFs (Fig. 5c). These data indicate that miR-495 directly regulates NOD1 expression through 3’UTR sequence binding.

### Effects of miR-495 on the high glucose-induced NF-κB and TGF-β1/Smad signaling pathways

**Fig. 6:**
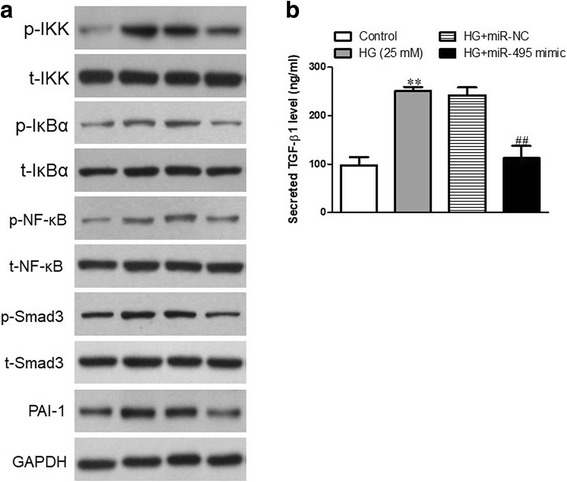
The effects of miR-495 on high glucose-induced NF-κB and TGF-β1/Smad signaling pathways. CFs were transfected with miR-495 mimic for 48 h, and then treated with 5.5 or 25 mM glucose for 24 h. **a** The protein expressions of p-IKK, t-IKK, p-IκBα, t-IκBα, p-NF-κB, t-NF-κB, p-Smad3, t-Smad3 and PAI-1 were determined via western blot. **b** The secreted TGF-β1 proteins in the supernatants of CFs were detected using ELISA assays. All data are presented as means ± SEM (*n* = 4). **p < 0.01 vs. control; ^##^*p* < 0.01 vs. vehicle + high glucose. The vehicle is transfection reagent alone

It is well established that NOD1 stimulation induces NF-κB activation in various biological systems [18, 19]. Based on our results, miR-495 could effectively prevent high glucose-induced pro-inflammatory and pro-fibrotic effects in human CFs, so the high glucose-stimulated NF-κB and TGF-β1/Smad signaling pathways were analyzed in human CFs. Our data show that introducing miR-495 obviously decreased the high glucose-induced expressions of p-IKK/t-IKK, p-IκBα/t-IκBα and p-NF-κB/t-NF-κB (Fig. 6a).

As fibroblasts are the primary source of TGF-β in the heart and might modulate the production of extracellular matrix [20], we analyzed the TGF-β pathway in CFs treated with miR-495 and incubated with high glucose. Our results show that the expressions of TGF-β1, p-Smad3 and PAI-1 were significantly higher after treatment with high glucose, whereas overexpression of miR-495 could effectively block the effect of high glucose (Fig. 6a, b).

## Discussion

Diabetes mellitus contributes to the aggravation of cardiovascular diseases. Cardiac fibrosis is one of the complications associated with diabetes, with a major role in multiple cardiac diseases, including cardiomyopathy, atrial fibrillation and myocardial infarction [21].

TGF-β, a pleiotropic cytokine, has an important role in multiple critical biological processes. TGF-β1 was considered to be involved in cardiac fibrosis associated with diabetes. MicroRNAs (miRNAs) are important regulators involved in multiple biological processes, and several of them play critical roles in cardiac fibrosis [22–26]. For example, miR-155 deficiency can prevent cardiac fibrosis in diabetic mice and attenuate the collagen synthesis induced by high glucose in mouse CFs [24]. Overexpression of miR-21 promotes cardiac fibrosis after myocardial infarction by directly targeting Smad7 [25]. Introduction of miR-30e attenuates isoproterenol-induced cardiac fibrosis by suppressing Snai1/TGF-β signaling [26].

In this study, we showed that high glucose increased NOD1 expression and decreased the level of miR-495 in human CFs. Overexpression of miR-495 attenuated high glucose-induced CF inflammation and differentiation into myofibroblasts, downregulated MMP expressions, and reduced collagen production through inhibition of the NF-κB and TGF-β1/Smad signaling pathways by directly targeting NOD1. To the best of our knowledge, it is the first report to clarify the effects of miR-495 on the function of CFs exposed to high glucose.

The differentiation of fibroblasts into myofibroblasts strongly increases in the myocardium of failing hearts. This is characterized by the expression of α-SMA and increased formation of disorganized collagen matrix [27]. Here, we also found that high glucose (5.5 mM) treatment could promote differentiation of CFs into myofibroblasts, which was consistent with the results previous studies [28]. The data also indicate that overexpression of miR-495 attenuated differentiation of CFs into myofibroblasts. These results suggest that miR-495 might play an important in high glucose-induced ECM remodeling by activating the differentiation of fibroblasts into myofibroblasts.

MMPs are important to maintain and degrade the ECM, which is part of the process of cardiac remodeling [29]. Previous reports have shown that MMP-2 and MMP-9 activities were significantly upregulated in diabetic heart, and inhibition of MMP activities is considered to have a cardioprotective role in diabetes [29, 30].

In our study, we demonstrated that MMP-2 and MMP-9 levels in CFs increased significantly in response to high glucose, and that was reversed by overexpression of miR-495 and further increased by inhibition of miR-495. These results showed a link between miR-495 and MMPs in cardiac fibrosis related to diabetes.

Collagen I and III are the main molecules of the ECM. They form fibrils and provide connective material and other structures in the myocardium [31]. Collagen accumulation contributes to the development of heart dysfunction. We found that high glucose promoted collagen I and III synthesis in CFs, and that this was partially inhibited by overexpression of miR-495.

A previous study reported that CF differentiation and dysregulation of MMPs could lead to the abnormal collagen deposition, which contributed to cardiac dysfunction in diabetic mice [28]. However, miR-495 played a protective role when introduced to CFs that were incubated with high glucose in vitro. Our findings suggest a critical role for miR-495 in modulating ECM remodeling in CFs exposed to high glucose.

NOD proteins are members of the NLR family, and they can induce specific inflammatory responses [32, 33]. Many of the diseases accompanied by high NOD1 activity are chronic inflammatory disorders, such as asthma and atopic eczema, emphasizing the critical role of this receptor in regulating the immune response [34, 35]. NOD1 is known to be involved in cytokine production, NF-κB activation and, interestingly, the induction of apoptosis [36, 37]. Moreover, it has been reported that the NOD1 agonist induced profound cardiac dysfunction, together with the activation of the NF-κB and TGF-β pathways in both cardiomyocytes and cardiac fibroblasts [11].

Two pro-inflammatory cytokines, COX2 and NOS2, are targets of the NF-κB pathway [3, 11]. A previous study reported that through Smad activation, TGF-β signals give rise to the expression of TGF-β target genes, including PAI-1, which is considered a critical regulator of tissue remodeling. Increases in PAI-1 level has been associated with cardiac fibrosis [38].

In this study, we found that high glucose-induced cardiac fibrosis was closely related to upregulation of NOD1 expression in vitro. Moreover, the high glucose-induced NOD1 expression in CFs was downregulated by miR-495 overexpression. Importantly, miR-495 overexpression could significantly inactivate the high glucose-induced NF-κB and TGF-β signaling pathways by downregulating the levels of p-IKK/t-IKK, p-IκBα/t-IκBα, p-NF-κB/t-NF-κB and p-Smad3/t-Smad3. Also, the expressions of COX2, NOS2, TGF-β1 and PAI-1 induced by high glucose were markedly reduced after overexpression of miR-495.

These results confirm that upregulation of miR-495 can protect CFs from high glucose-induced cardiac fibrosis through the modulation of the NF-κB and TGF-β signaling pathways by directly targeting NOD1.

## Conclusions

Our results show that the introduction of miR-495 ameliorates high glucose-induced inflammatory reactions, cell differentiation and extracellular matrix accumulation of human CFs via the modulation of the NF-κB and TGF-β1/Smad signaling pathways by downregulation of NOD1 expression. These findings provide further evidence for the protective effect of miR-495 overexpression on high glucose-induced cardiac fibrosis.

## Abbreviations

CFs: Cardiac fibroblasts
DM: Diabetes mellitus
ECM: Extracellular matrix
miRNA: microRNA
MMP: Matrix metalloproteinase
